# Gymnemic Acids Inhibit Adhesive Nanofibrillar Mediated *Streptococcus gordonii–Candida albicans* Mono-Species and Dual-Species Biofilms

**DOI:** 10.3389/fmicb.2019.02328

**Published:** 2019-10-11

**Authors:** Raja Veerapandian, Govindsamy Vediyappan

**Affiliations:** Division of Biology, Kansas State University, Manhattan, KS, United States

**Keywords:** bacteria–fungi interactions, *Candida albicans*, *Streptococcus gordonii*, nanofibrils, gymnemic acid, biofilm inhibition, GAPDH, mixed oral biofilms

## Abstract

Dental caries and periodontitis are the most common oral disease of all age groups, affecting billions of people worldwide. These oral diseases are mostly associated with microbial biofilms in the oral cavity. *Streptococcus gordonii*, an early tooth colonizing bacterium and *Candida albicans*, an opportunistic pathogenic fungus, are the two abundant oral microbes that form mixed biofilms with augmented virulence, affecting oral health negatively. Understanding the molecular mechanisms of the pathogen interactions and identifying non-toxic compounds that block the growth of biofilms are important steps in the development of effective therapeutic approaches. In this *in vitro* study we report the inhibition of mono-species or dual-species biofilms of *S. gordonii* and *C. albicans*, and decreased levels of biofilm extracellular DNA (eDNA), when biofilms were grown in the presence of gymnemic acids (GAs), a non-toxic small molecule inhibitor of fungal hyphae. Scanning electron microscopic images of biofilms on saliva-coated hydroxyapatite (sHA) surfaces revealed attachment of *S. gordonii* cells to *C. albicans* hyphae and to sHA surfaces via nanofibrils only in the untreated control, but not in the GAs-treated biofilms. Interestingly, *C. albicans* produced fibrillar adhesive structures from hyphae when grown with *S. gordonii* as a mixed biofilm; addition of GAs abrogated the nanofibrils and reduced the growth of both hyphae and the biofilm. To our knowledge, this is the first report that *C. albicans* produces adhesive fibrils from hyphae in response to *S. gordonii* mixed biofilm growth. Semi-quantitative PCR of selected genes related to biofilms from both microbes showed differential expression in control vs. treated biofilms. Further, GAs inhibited the activity of recombinant *S. gordonii* glyceraldehyde-3-phosphate dehydrogenase (GAPDH). Taken together, our results suggest that *S. gordonii* stimulates the expression of adhesive materials in *C. albicans* by direct interaction and/or signaling, and the adhesive material expression can be inhibited by GAs.

## Introduction

Dental caries is a polymicrobial biofilm-induced disease affecting 3.5 billion people globally (Kassebaum et al., 2017). The worldwide annual total costs due to dental diseases are estimated to be around $545 billion in 2015 (Righolt et al., 2018). New therapeutic approaches are required to manage these biofilm-associated oral diseases. We need an efficient antimicrobial agent which inhibits biofilm formation, while not exerting selective pressure on the oral microbiome. *Candida albicans* is a fungus that is the etiologic agent of oral thrush and denture stomatitis, two mucosal oral biofilm infections that particularly affect immunocompromised patients and elderly people, respectively (Odds, 1987). *C. albicans* and *Streptococcus* bacterial species are abundant in the oral cavity and readily form mixed biofilms which are resistant to antimicrobials and serve as a source for systemic infections (Dongari-Bagtzoglou et al., 2009; Silverman et al., 2010; Diaz et al., 2012; Ricker et al., 2014; O’Donnell et al., 2015). Some of the streptococci (e.g., *Streptococcus mutans*) are the causative agents of dental caries and gum disease. Recent studies have shown that a complex interaction and aggregation occurs between streptococci and *C. albicans*, and the molecular mechanisms are poorly understood (Dutton et al., 2014; Hwang et al., 2017).

*Candida albicans* is a commensal and an opportunistic human fungal pathogen found in cutaneous, oral, intestinal, and genital regions, and can initiate various forms of Candidiasis. Various groups of oral bacteria are shown to interact with *C. albicans* and influence the disease severity (Dongari-Bagtzoglou et al., 2009; Harriott and Noverr, 2011). Oral streptococcal species, including *Streptococcus gordonii, Streptococcus oralis*, and *S. mutans*, interact with *C. albicans* and augment both fungal and bacterial virulence (Silverman et al., 2010; Ricker et al., 2014; O’Donnell et al., 2015; Hwang et al., 2017). Other bacteria, including *Staphylococcus aureus* (Harriott and Noverr, 2009) and *Acinetobacter baumannii* (Uppuluri et al., 2018), use *C. albicans* hyphae as a substratum for attachment, and form robust biofilms.

*Candida albicans* exists in yeast, pseudohyphae, and hyphal growth forms. The transition from yeast or pseudohyphae to hyphae is required for its tissue invasion and biofilm formation. Mutants that are defective in hyphal growth are avirulent and unable to form biofilms (Lo et al., 1997; Nobile and Mitchell, 2006). Hence, *C. albicans* hyphae play a pivotal role in biofilm growth and virulence. Some of the oral bacteria, including *S. gordonii* are shown to promote the hyphal growths of *C. albicans* and bind preferably to these hyphal surfaces (Bamford et al., 2009). This hyphal binding increases biofilm mass, and chemical inhibition of candida hyphae reduces biofilm mass (Bamford et al., 2009). Several bacterial pathogens exploit *C. albicans* hyphae for their attachments (Silverman et al., 2010; Diaz et al., 2012; Dutton et al., 2014; Xu et al., 2014b; O’Donnell et al., 2015). A recent study has shown that yeast cells of *Candida glabrata* bind to *C. albicans* hyphae and form fungal-fungal biofilms in the oral milieu (Tati et al., 2016). Microbial biofilms are highly resistant to antimicrobial agents, sequestering them and causing tissue inflammation (Nett et al., 2010; Vediyappan et al., 2010; Xu et al., 2014b). It is plausible that inhibiting *C. albicans* hyphal growth with non-toxic small molecules could abrogate the hyphae-related virulence, including *C. albicans* interaction with bacteria and the growth of polymicrobial biofilms.

Gymnemic acids (GAs), a family of triterpenoid molecules from the medicinal plant *Gymnema sylvestre*, were shown to block *C. albicans* yeast-to-hypha transition and hyphal growth *in vitro* and in a worm (*Caenorhabditis elegans*) model of invasive candidiasis (Vediyappan et al., 2013). GAs contain various pharmacological properties, including antagonistic activity against the β-isoform of Liver-X-Receptor (LXR) which could result in decreased lipid accumulation in liver cells (Renga et al., 2015), suppressing sweet taste sensation by binding to taste receptors, T1R2, and T1R3 (Sanematsu et al., 2014), and blocking the uptake of glucose in the intestinal cells (Wang et al., 2014). The GA-rich gymnema extract has been used in humans to treat diabetes and obesity (Baskaran et al., 1990; Porchezhian and Dobriyal, 2003; Leach, 2007). A recent clinical study confirmed the traditional use of *G. sylvestre* for diabetes (Zuniga et al., 2017). Since *S. gordonii* and other oral bacteria use *C. albicans* hyphae for their attachment (Bamford et al., 2009) and GAs block the hyphal growth of *C. albicans*, we wanted to test the hypothesis that prevention of *C. albicans* hyphal growth using GAs could abolish bacteria *– C. albicans* interactions and the formation of mixed biofilms. In the current study, we show a synergistic interaction between *S. gordonii* and *C. albicans in vitro*, and the addition of gymnemic acids (GAs) prevented the growth of mono-species or dual-species biofilms. Our results show, for the first time to our knowledge, formation of “nanofibrillar” structures from *C. albicans* hyphae in response to *S. gordonii* co-culture, which correlates with their enhanced interaction and biofilms growth. Treating mono-species or dual-species biofilms with GAs abolished these structures and reduced biofilm growth.

## Materials and Methods

### Strains, Culture Conditions, and Compounds

*Streptococcus gordonii* ATCC 10558 (generously provided by Dr. Indranil Biswas, Kansas University Medical Center, Kansas City, KS) and *C. albicans* SC5314 (genome sequenced) were used to generate mono-species or dual-species biofilms in 24-well microtiter plates (TPP, Cell culture treated) under static condition. *Escherichia coli* 10-Beta (NEB) and BL21(DE3) (Novagen, Madison, WI, United States) were used for cloning and expression of recombinant protein, and were routinely grown in Luria-Bertani (LB) broth or on LB agar. GAs were purified from *G. sylvestre* plant leaf extract, obtained from Suan Forma Inc., NJ, United States, according to the published protocols (Vediyappan et al., 2013; Sanematsu et al., 2014) and a mixture of GAs was used in this study. The GA mixture contains at least five species (GA-III, GA-IV, GA-XIII, GA-XIV, and GA-I) and therefore, the term GAs was used throughout in the text. All five GA species that we used have similar bioactivities of yeast-to-hypha inhibition (Vediyappan et al., 2013; Sanematsu et al., 2014). We have isolated GA-I recently and it was included in the GA mixture. There are 18 different species of GA that have been reported (Liu et al., 1992; Porchezhian and Dobriyal, 2003; Di Fabio et al., 2013). The GA mixture (50 mg/ml) was solubilized as a stock solution in TYES growth medium, filter sterilized (0.4 μm syringe filter), and diluted in the growth medium as required.

### Determination of Minimum Biofilm Inhibition Concentrations (MBICs) and Growth Kinetics

*Streptococcus gordonii* and *C. albicans* co-exist in the oral cavity as abundant microbes, and the former is known to attach to the hyphal surfaces of the latter, forming a mixed-species biofilm with enhanced virulence. Preventing the growth of these biofilms by non-toxic small molecules would limit oral diseases and their systemic dissemination. Since GAs are known to inhibit *C. albicans* hyphal growth, we wanted to know if GAs can inhibit *S. gordonii* and *C. albicans* biofilms. First, we wanted to determine the MBIC of GAs against these microbial biofilms. The MBIC is the lowest concentration of GAs that inhibit maximum amount of biofilm growth. MBIC was determined in 24-well plates as previously described (Saputo et al., 2018), with slight modifications using TYES broth medium (1% tryptone and 0.5% yeast extract at pH 7.0 with 1% (wt/vol) sucrose). Briefly, suspensions of *S. gordonii* (∼2 × 10^6^ CFU/ml) or *C. albicans* yeast cells (2 × 10^4^ CFU/ml), according to Kim et al. (2017), were added into 24-well plates containing serially diluted GAs at concentrations ranging from 0 to 600 μg/mL. The plates were incubated at 37°C with 5% CO_2_ for 18 h statically. Medium alone and medium with GAs were also included in parallel as blanks to rule out that the observed readings were not due to precipitation of GAs or non-specific absorbance. After washing off unbound cells and medium with PBS, the adhered biofilms were stained with crystal violet (0.1%, CV solubilized in water) solution (Merritt et al., 2005). After removing the unbound CV, the wells were washed (at least two times) with PBS and dried to remove residual buffer. Biofilm attached CV stains were solubilized in 95% ethanol, and the absorbance was measured at 595 nm with a Victor 3 multimode reader (Perkin Elmer, United States). Experiments were repeated at least three times, each with triplicates, and representative results are shown.

To determine the effect of GAs on the growth rates of *S. gordonii* and *C. albicans*, we used a Bioscreen-C real time growth monitoring system (Oy Growth Curves Ab Ltd., Finland). In this method, 200 μl of growth medium containing exponentially growing *S. gordonii* or *C. albicans* yeast cells (each at *A*_600_ = 0.1) were added into the honeycomb wells (triplicate) with GAs (400, 500, and 600 μg/mL in 200 μl total volume) or without GAs (control), and their growth rates were measured for 24 h. The plates were incubated at 37°C without shaking except for 10-s of shaking before reading absorbance at 600 nm at 30-min intervals. The overall objective of the kinetic growth readings of *S. gordonii* and *C. albicans* in the presence or absence of GAs was to determine if GAs exert any toxic effect on the microbes.

### Unstimulated Whole Saliva Preparation

Human saliva collection and processing were done as described previously (Jack et al., 2015). Briefly, unstimulated whole human saliva was collected from six healthy volunteers with Institutional Review Board (IRB) protocol approval (#9130.1) from Kansas State University. All the subjects gave written informed consent approved by the IRB committee. Saliva was pooled and mixed with 2.5 mM dithiothreitol and kept in ice for 10 min before clarification by centrifugation (10,000 × *g* for 10 min). The supernatant was diluted to 10% in distilled water and filter sterilized through a 0.22-μm nitrocellulose filter and stored at −80°C in aliquots. Diluted saliva was used to coat the microtiter wells and hydroxyapatite (HA) disks (Clarkson Chromatography Products, PA, United States) overnight.

### Mono-Species and Dual-Species Biofilm Assay

To test the effect of GAs on biofilm formation in saliva-coated wells and on hydroxyapatite (sHA) disks, *S. gordonii* and *C. albicans* were grown alone or in combination in TYES medium with or without GAs (500 μg/ml) statically for 18 h at 37°C and in 5% CO_2_, as reported with some minor modifications (Dutton et al., 2014; Ricker et al., 2014). Two types of biofilm models were used. (i) Biofilms grown on saliva-coated hydroxyapatite (sHA) disks that were used for Scanning Electron Microscopic (SEM) analysis, and (ii) biofilms grown on the bottom of the saliva coated polystyrene microplates (24-wells, TPP cell culture treated). The biofilms developed on the microplate surfaces were used for CV staining, RNA, and for eDNA isolations. Briefly, sHA disks were placed in a 24-well plate and inoculated with approximately 2 × 10^6^ (CFU/ml) of *S. gordonii* or/and 2 × 10^4^ (CFU/ml) of *C. albicans* according to Kim et al. (2017) in the TYES medium with or without GAs. The effect of GAs against biofilm formation in the microtiter wells was determined using CV staining (Merritt et al., 2005), as described above (MBIC section). Experiments were repeated at least three times each with triplicates, and representative results are shown.

### Measurement of Biofilm Extracellular DNA (eDNA)

Extracellular DNA was measured as described by Jack et al. (2015). Briefly, biofilms were scraped from saliva-coated wells into 0.5 mL TE buffer (10 mM Tris–HCl, pH 7.5, 1 mM EDTA), sonicated for 15 s at low speed (20 pulse, Branson Ultrasonic 250), and the cell-free DNA was collected by centrifugation at 10,000 × *g* for 5 min. The DNA concentration was then analyzed from the supernatant using a NanoDrop 2000 spectrophotometer (Thermo Scientific, United States).

### Scanning Electron Microscopy (SEM)

Scanning Electron Microscopy was done as per the standard protocol described previously (Erlandsen et al., 2004). Briefly, sHA disks with biofilms on their surfaces were fixed with 2% paraformaldehyde and 2% glutaraldehyde in 0.15 M sodium cacodylate buffer, pH 7.4, containing 0.15% Alcian blue. Biofilms grown on sHA disks were washed with 0.15 M cacodylate buffer and dehydrated in a graded series of ethanol concentrations. Specimens were mounted on adhesive carbon films and then coated with 1 nm of platinum using an Ion Tech argon ion beam coater. Prepared samples were observed in a SEM (Field Emission Scanning Electron Microscope, Versa 3D Dual Beam, Nikon).

### RNA Isolation, cDNA Synthesis, and Semi-Quantitative RT–PCR

Biofilms grown in 24-well microtiter plates were treated with RNAprotect bacteria reagent (Qiagen, Valencia, CA, United States) for 5 min to stabilize RNA, and stored at −80°C. Total RNA was isolated from the biofilms using the TRIzol reagent (Invitrogen, Carlsbad, CA, United States) as per manufacturer instructions. The concentration of RNA was determined by measuring the *A*_260_ in a NanoDrop 2000 spectrophotometer (Thermo Scientific, United States). Total RNA (1 μg) was reverse transcribed into cDNA using the SuperScript III indirect cDNA labeling kit (Invitrogen), as per the manufacturer’s instructions with slight modifications. The semi-quantitative RT-PCR using 2X PCR Master Mix (Promega Corporation, Madison, WI, United States) and primers was carried out in a 20 μL reaction volume (1 μL cDNA, 10 μL Master Mix, 0.5 μM of each primer). Primers were designed using PrimerQuest^®^ (Integrated DNA Technologies), and the details are given in Tables 1, 2. The internal control was *16S rRNA* for *S. gordonii* and *TDH3* for *C. albicans*. The cycling conditions consisted of initial denaturation at 94°C for 3 min followed by denaturation at 94°C for 30 s, annealing at 50 or 58°C for 30 s, and extension at 72°C for 45 s, then final extension at 72°C for 7 min. Twenty microliters of each PCR product was electrophoresed on an agarose gel (1.2% w/v) containing ethidium bromide (0.5 μg/ml). Images of the amplified products were acquired with an Alpha Imager; the intensity was quantified using the Image J software (NIH, United States). The band intensity was expressed as mRNA fold expression [specific gene expression/internal control gene (*16S rRNA* or *TDH3*)]. The intensity of each DNA band in the control cells was determined, taken as 1, and compared with the respective treated group (Sivaprakasam et al., 2016).

**TABLE 1:** List of *S. gordonii* specific primers used for semi-quantitative RT-PCR.

**Gene name**	**Description**	**Direction**	**Sequence (5′-3′)**	**Product Size (bp)**
*csh*A	Cell surface hydrophobicity	Forward	GACAAGCAGTTCGTTGGTAAAC	264
		Reverse	GGTTCCTTGACCTGGAATAGAC	
*ldh*	Lactate dehydrogenase	Forward	CGTTCAGTTCACGCCTACAT	328
		Reverse	CAGCTGGTTGACCGATAAAGA	
*gapdh*	Glyceraldehyde-3-phosphate dehydrogenase	Forward	CTCGCATCAACGACCTTACA	557
		Reverse	AGCAGCACCAGTTGAGTTAG	
*gftG1*	Glucosyltransferase G	Forward	CCATCCCTTGAGTACGAGTTTC	564
		Reverse	GTGGAGTAGAGCCAACGATTAC	
*scaA*	Metal ABC transporter substrate-binding lipoprotein	Forward	GGGAATATC TTGGCGGTACAA	288
		Reverse	GGTCTTGAGACTCTTGGCATAG	
*scaR*	Iron-dependent transcriptional regulator	Forward	TAGTCCACCATCTGGGCTATAC	281
		Reverse	GCCAACTTGAAGGCCATTTC	
*16S rRNA*	16S ribosomal RNA	Forward	CCATAGACTGTGAGTTGCGAAC	427
		Reverse	CCGTCCCTTTCTGGTAAGATAC	

**TABLE 2:** List of *C. albicans* primers used for semi-quantitative RT-PCR.

**Genename**	**Description**	**Direction**	**Sequence (5′-3′)**	**Product Size (bp)**
*CSH1*	Cell surface hydrophobicity	Forward	GCTGTCGGTACTATGAGATTGG	245
		Reverse	CTGTCTTCTGCGTCGTCTTT	
*ZRT1*	Zinc-regulated transporter	Forward	ATGCCCGTGATACTGGAAAG	312
		Reverse	GGGTGATCAATGCAAACATGAG	
*NRG1*	Transcription factor/co-repressor	Forward	ACTACAACAACCTCAGCCATAC	254
		Reverse	CAAGGGAGTTGGCCAGTAAA	
*PRA1*	pH-regulated antigen	Forward	CGCTGACACTTATGAGGAAGTC	258
		Reverse	CTAGGGTTGCTATCGGTATGTTG	
*TDH3*	Glyceraldehyde-3-phosphate dehydrogenase	Forward	GTCGCCGTCAACGATCC	455
		Reverse	GTGATGGAGTGGACAGTGGTC	

### Cloning, Expression, and Purification of rGAPDH

Glyceraldehyde-3-phosphate dehydrogenase is reported to be present in various streptococcal cell surfaces which mediates cell adhesion and plays an important role in bacterial infection and invasion (Brassard et al., 2004; Jin et al., 2011; Wang et al., 2012). For example, *S. gordonii* cell surface GAPDH binds to the FimA protein of Porphyromonas *gingivalis* and forms a mixed biofilm (Maeda et al., 2004a, b). GAPDH has multiple functions in various organisms (Sirover, 2017). Further, our semi-quantitative RT-PCR results showed reduced transcripts of *gapdh* in both mono-species and mixed-species biofilms, and hence we pursued to analyze the role of this protein. GAPDH gene from *S. gordonii* was PCR amplified using primers GAPDH-F (5′-ATTCCATATGGTAGTTAAAGTTGGTATTAACGGT-3′) and GAPDH-R (5′-GCGCTCGAGTTTAGCGATTTTCGCGAA GTATTCAAG-3′), where the underlined sequences in the forward and in reverse primers indicate *Nde*I and *Xho*I restriction sites, respectively. Following PCR amplification of chromosomal DNA from *S. gordonii* strain ATCC 10558, amplicons of 1008 bp were digested with *Nde*I and *Xho*I and inserted into the predigested pET28b plasmid (Novagen, Madison, WI, United States). Successful cloning of the gene was confirmed by restriction endonuclease and DNA sequence analyses (Supplementary Figures S1, S2). Recombinant plasmid was transformed into *E. coli* BL21 (DE3) for overexpression. Expression of GAPDH-6His protein was induced with 1 mM isopropyl-β-d-thiogalactopyranoside (IPTG) when cultures reached an optical density at 600 nm (OD600) of 0.6, and cells were harvested after 4 h. The cell pellet from 2 L of culture was resuspended in 40 ml of a buffer containing 50 mM NaH_2_PO_4_ pH 8.0, 300 mM NaCl, 20 mM imidazole with 1× protease inhibitor cocktail (Roche) and 1 mM phenylmethylsulfonyl fluoride (PMSF), and cells were lysed by French press (∼19,000 psi). The lysate was centrifuged at 10,000 × *g* for 20 min at 4°C. Recombinant His-tagged GAPDH was purified using Ni-NTA Agarose (Qiagen, Valencia, United States) in native conditions according to the manufacturer’s recommendations. GAPDH was eluted using gradients of increasing imidazole concentration (100–300 mM). Fractions containing rGAPDH were pooled and dialyzed against distilled water and used for subsequent analysis.

### SDS-PAGE and Immunoblotting

The purity of the proteins was checked using SDS-PAGE electrophoresis in a vertical electrophoretic mini-cell unit (Bio-Rad, Hercules, CA), in Tris-glycine running buffer (25 mM Tris, 192 mM glycine, 0.1% SDS [pH 8.3]), for 1 h at 120 V. Proteins were transferred to an Immobilon-P PVDF membrane (pore size, 0.45 μm; Millipore Sigma, United States) and blocked with 5% non-fat dry milk in Tris-buffered saline (20 mM Tris, 150 mM NaCl, 0.2% Tween 20 [pH 7.5]). Membranes were incubated with anti-*Sg*GAPDH immune sera raised in rabbits, followed by incubation with secondary antibody (anti-rabbit IgG; Cell Signaling Technology, United States). Reacted protein bands were visualized by using Pierce^TM^ ECL 2 Western Blotting Substrate (Thermo Scientific, United States) and imaging.

### Determination of GAPDH Activity

Glyceraldehyde-3-phosphate dehydrogenase activity was measured in the presence and absence of GAs using Glyceraldehyde 3 Phosphate Dehydrogenase Activity Colorimetric Assay Kit (ab204732, Abcam, Cambridge, MA, United States) as per the manufacturer’s instructions. Briefly, purified rGAPDH protein (0.1 μM) was mixed with and without GAs (100 and 200 μM), followed by the addition of reaction mix supplied from the kit components. The conversion of NAD to NADH was monitored every 10 s spectrometrically using a Victor 3 multimode reader (Perkin Elmer, United States) at 450 nm.

### Statistical Analysis

Data from multiple experiments (≥3) were quantified and expressed as mean ± SD, and differences between groups were analyzed using one-way ANOVA. Tukey multiple comparison test was used to analyze significance among the groups. *p* ≤ 0.05 was considered significant in all analyses. The data were computed with GraphPad Prism version 7.0 software.

## Results

### Determination of Minimum Biofilm Inhibition Concentration

**FIGURE 1:**
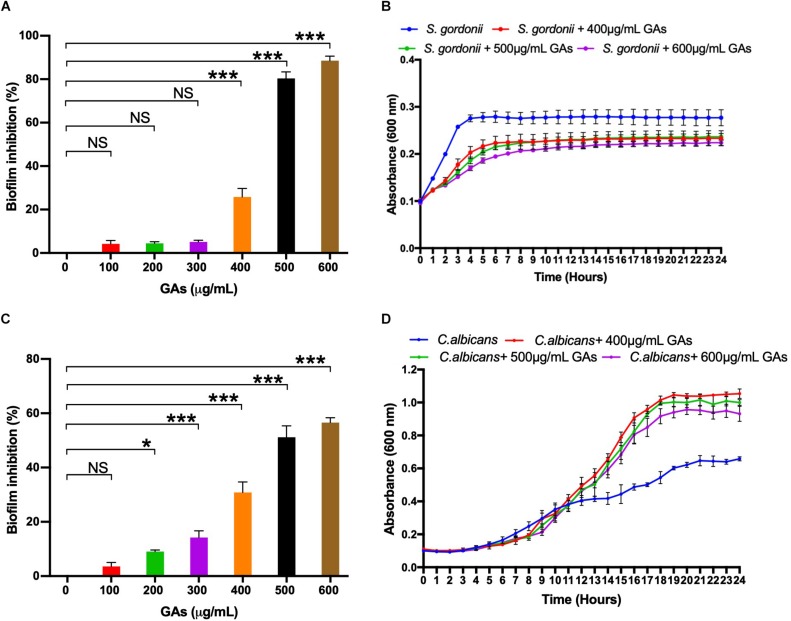
Determination of minimum biofilm inhibition concentration (MBIC) of GAs against *S. gordonii* **(A)** and *C. albicans* **(C)**. Varying concentrations of GAs (0–600 μg/ml) were used in TYES medium containing *S. gordonii* or *C. albicans* in 24-wells with triplicates and incubated at 37°C with 5% CO_2_ statically for 18 h. Biofilms grown without GAs served as controls. Inhibition of biofilm growth was analyzed by CV staining and % inhibition of biofilm was calculated. The results represent means ± standard deviations for three independent experiments. Statistical significance was determined by ANOVA and a Dunnett’s multiple comparison test. ^∗^*p* < 0.05, ^∗∗∗^*p* < 0.0001, NS, not significant. Analysis of planktonic growths of *S. gordonii* **(B)** and *C. albicans* **(D)** with and without GAs. Honeycomb wells containing *S. gordonii* or *C. albicans* in 200 μl TYES medium with or without GAs were used to monitor their growth rates in a Bioscreen-C system for 24 h. Three different concentrations of GAs (400, 500, 600 μg/ml) were used. Absorbance was recorded every 30-min intervals at 600 nm as described in the section “Materials and Methods.” The results represent means ± standard deviations for three independent experiments.

To determine the minimum amount of GAs needed to inhibit maximum biofilm growth of *S. gordonii* and *C. albicans*, an MBIC assay was performed with increasing concentrations of GAs (0–600 μg/mL). Biofilms were quantified by CV staining, and the results showed a concentration-dependent antibiofilm activity of GAs against *S. gordonii* and *C. albicans* (Figures 1A,C, respectively). A significant inhibition of biofilm formation was found from concentrations >400 μg/mL for *S. gordonii* and from concentrations >200 μg/mL for *C. albicans*. While maximum biofilm growth inhibition (80–90%) was found between 500 and 600 μg/mL for *S. gordonii*, about 50% biofilm growth was inhibited for *C. albicans* at that GAs concentration (Figure 1C).

### Impact of GAs on the Growth Kinetics of *S. gordonii* and *C. albicans*

To determine if GAs are toxic to *S. gordonii* and *C. albicans*, we measured their growth kinetics as planktonic cells in the presence or absence of GAs using Bioscreen-C growth monitor at 37°C in TYES medium, as described in the section “Materials and Methods.” Since GAs inhibited *S. gordonii* biofilm growth from a concentration of 400 μg/ml upward, we used three different concentrations of GAs (400, 500, and 600 μg/mL) to assess its effects. As shown in Figure 1B, the growth of *S. gordonii* is slightly reduced in the presence of GAs. When *S. gordonii* was exposed to 400 and 500 μg/mL, GAs showed only slight growth inhibition, while GAs at 600 μg/mL affected *S. gordonii* growth to a greater extent. In contrast, GAs did not affect *C. albicans* growth rate until 12 h. At that point, GAs (400, 500, and 600 μg/mL) promoted the growth of *C. albicans* (Figure 1D), and each concentration had a similar effect. The mechanism for the growth induction is not known. One possibility may be that GAs may interfere with the carbohydrate metabolism of *C. albicans*, and as an adaptive response, the fungus turns on a different metabolic pathway for its cellular energy needs, resulting in an increased growth rate compared to the control. This conjecture is based on the fact that GAs are used for treating metabolic diseases in humans (e.g., lowering plasma glucose in diabetes) (Baskaran et al., 1990; Leach, 2007). Further studies on biofilm gene expression in the presence of GAs and biochemical validation are warranted. Taken together, GAs inhibited the growth of *S. gordonii* slightly at 400–600 μg/mL but did not inhibit *C. albicans’* growth under the conditions used. Since 500 μg/ml GAs maximally inhibited biofilms of both microbes with minimal impacts on their growth rates, we employed this concentration (500 μg/mL) throughout the study to determine its effect on mono-species or dual-species biofilms.

### Inhibition of *S. gordonii* and *C. albicans* Mono-Species and Dual-Species Biofilms Grown in 24-Well Microtiter Plates by GAs

**FIGURE 2:**
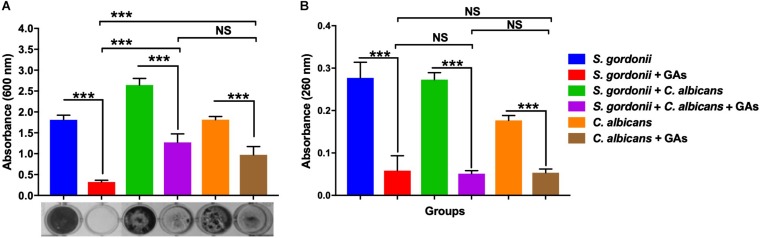
Effect of GAs on *S. gordonii* and *C. albicans* mono-species or dual-species biofilms. **(A)** Crystal violet staining of *S. gordonii* and *C. albicans*, either as mono-species or as dual-species biofilms with and without GAs in saliva coated 24-well plates. **(B)** Measurement of eDNA from mono-species and dual-species biofilms with and without GAs. eDNA from pooled biofilms was released by gentle sonication as mentioned in the methods. The results represent means ± standard deviations for three independent experiments. Data were analyzed by one-way ANOVA followed by Tukey’s multiple comparison test. NS, not significant; ^∗∗∗^*p* < 0.001.

The anti-biofilm efficacy of GAs was assessed under *in vitro* condition by measuring the binding of CV to *S. gordonii* biofilms cells grown in 24-well plates. The antibiofilm activity of GAs was effective at 500 μg/ml against *S. gordonii* and *C. albicans* mono-species and dual-species biofilms (Figure 2A). GAs treatment significantly reduced the amount of *S. gordonii* biofilms (Figure 2A). Similarly, the mixed biofilms were also reduced with GAs treatment, and are significant as analyzed by one-way ANOVA (*p* = 0.001). When Tukey multiple comparison test was used, the *S. gordonii* biofilm was significantly inhibited by GAs compared to *C. albicans* or dual-species biofilms (Figure 2A).

### Effective Reduction of eDNA in Mono-Species and Dual-Species Biofilm by GAs Treatment

Extracellular DNA is a part of the polymeric materials in the extracellular matrix of biofilms (Xu and Kreth, 2013). To examine the effects of GAs on biofilm eDNA, mono-species and dual-species biofilms were grown in TYES medium. High levels of eDNA were found in both mono-species and dual-species biofilms. Interestingly, a significant reduction in eDNA concentrations were observed in the biofilms treated with GAs (*p* = 0.001, Figure 2B). There was no significant difference in the amount of eDNA reduction among the three groups as determined by Tukey multiple comparison test (Figure 2B), suggesting GAs treatment affects eDNA in all these biofilms similarly.

### Inhibition of *S. gordonii* and *C. albicans* Mono-Species and Dual-Species Biofilms on sHA Disks

**FIGURE 3:**
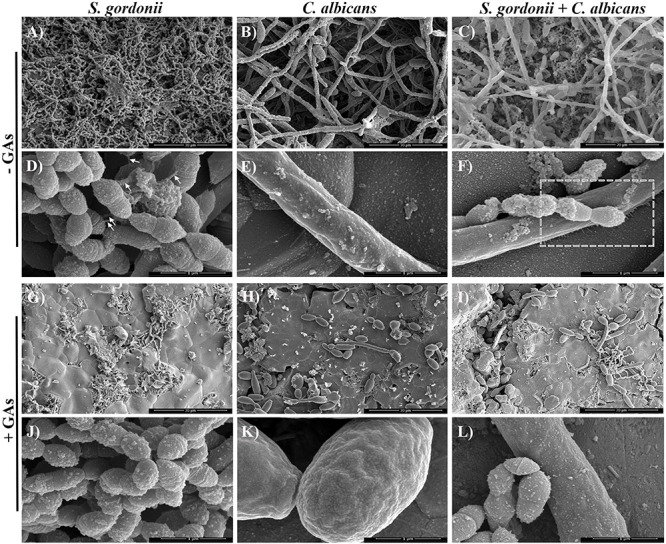
Scanning electron microscopy (SEM) observations of mono-species and dual-species biofilms grown on sHA in the presence or absence of GAs. Images of mono-species and dual-species biofilms grown for 18 h in the absence (control, **A–F**) and in the presence of GAs **(G–L)** at 500 μg/ml concentration. Magnifications: **(A–C,G–I)** scale bar 20 μm, and **(D–F,J–L)** scale bar 1 μm. Biofilms grown with GAs show few cells on the sHA surfaces compared to dense layers of cells with exopolysaccharides (EPS) in the control groups. Short fibrils in the control *S. gordonii* biofilms that are attached to neighboring cells are shown (**D**, arrows). Changes in the biofilm surface textures and absence of fibrils were observed in the GAs treated biofilm groups **(G–L)**. GAs treated *C. albicans* show mostly yeast or pseudohyphal cells with few hyphae **(H,K)**. Dashed box in **F** was further magnified in Figure 4 to show nanofibrillar structures. In GAs treated dual-species biofilms, weak or no fibrillar structures from *S. gordonii* and none from *C. albicans* were found **(J–L)**.

Scanning Electron Microscopy analysis was carried out to examine the structures of mono-species and dual-species biofilms formed on sHA disks that were treated with GAs. Biofilms formed on sHA disks were fixed, stained with Alcian blue, and processed as described (Erlandsen et al., 2004). These authors used different cationic stains to visualize bacterial surface structures by SEM. Since the microbial surface structures are negatively charged, the positively charged Alcian blue stain binds to the cell surface nanofibrils and improves their detection by SEM. SEM micrographs of *S. gordonii* revealed the formation of biofilms with thick aggregates of cells and patches of exopolysaccharide (EPS) on the surface of sHA (Figure 3A). Interestingly, very little biofilm of *S. gordonii* was found on the GAs treated-sHA disk, and large empty areas were seen mostly (Figure 3G). The SEM results are consistent with the results of the *in vitro* biofilm growth assay (Figure 2A). As expected, *C. albicans* control biofilms (B) contained multilayers of hyphae and in the GAs exposed biofilms, very little yeast and pseudohyphal cells were present on the sHA disks (H-I). It is worth mentioning that although GAs promote the growth rate of *C. albicans* (Figure 1D), the cells that grow are mostly planktonic yeast cells, and they poorly attach or fail to form biofilms. *S. gordonii* and *C. albicans* dual biofilms contained both dense bacterial and fungal hyphal cells (Figure 3C), and their abundance was decreased by GAs treatment (Figure 3I). The inhibitory effect of GAs was clearly demonstrated in the SEM micrographs of biofilms. Interestingly, mono-species and dual-species biofilms grown on sHA disks treated with GAs has no or few cell surface nanofibrils and instead exhibited smooth hyphal surfaces (Figures 3J–L).

### *S. gordonii* and *C. albicans* Co-culture Promotes Formation of Extracellular Fibrils

**FIGURE 4:**
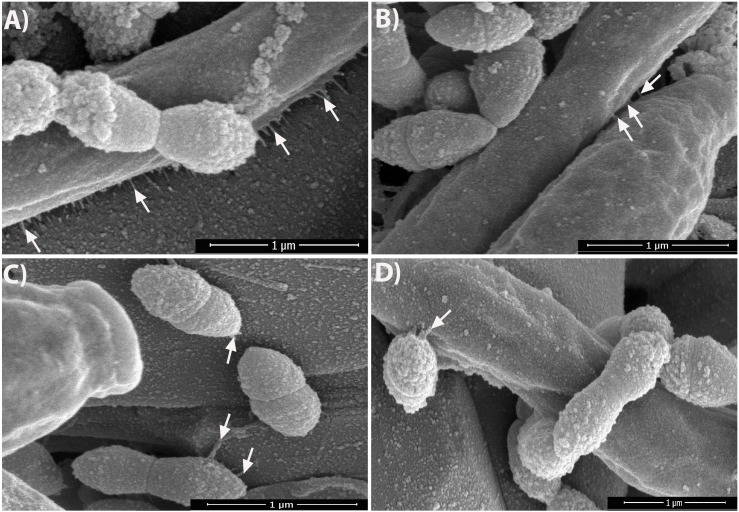
Dual-species biofilms in the absence of GAs promote nanofibrillar-mediated interactions. SEM images of dual-species biofilms showing nanofibrillar structures from *C. albicans* hypha that are attached to the sHA (**A**, arrows) as well as between two hyphae (**B**, arrows). *S. gordonii* exhibits high affinity to hypha by its fibrillar attachment and by tight coiling around the hypha **(D)**, and also to sHA **(C)** which mimics teeth. The images were zoomed to show the nanofibrils. Scale bar, 1 μm.

Viewing the biofilms at higher magnification (50,000× or 1 μm) revealed that, without GAs exposure, there were short fibrils between *S. gordonii* cells, and some of these fibrils were attached to sHA (Figure 4C, arrows). As expected, *C. albicans* biofilms without GAs treatment produced mostly hyphae. Interestingly, *C. albicans* biofilms co-cultured with *S. gordonii* (dual-species biofilm) without GAs showed several closely attached bacterial-fungal cells with extracellular materials (Figure 4A). Strikingly, we found several thin fibrils from hypha that are in close contact with the sHA disk (Figure 4A, arrows) or to the neighboring hypha (Figure 4D, arrows). *S. gordonii* was in close contact with the *C. albicans* hyphae as the bacterium coiled around the hypha, and also appeared to be directly attached with the help of fibrils (Figures 4C,D, arrows).

### Modulation of Gene Expression in Mono-Species and Dual-Species Biofilms With and Without GAs

**FIGURE 5:**
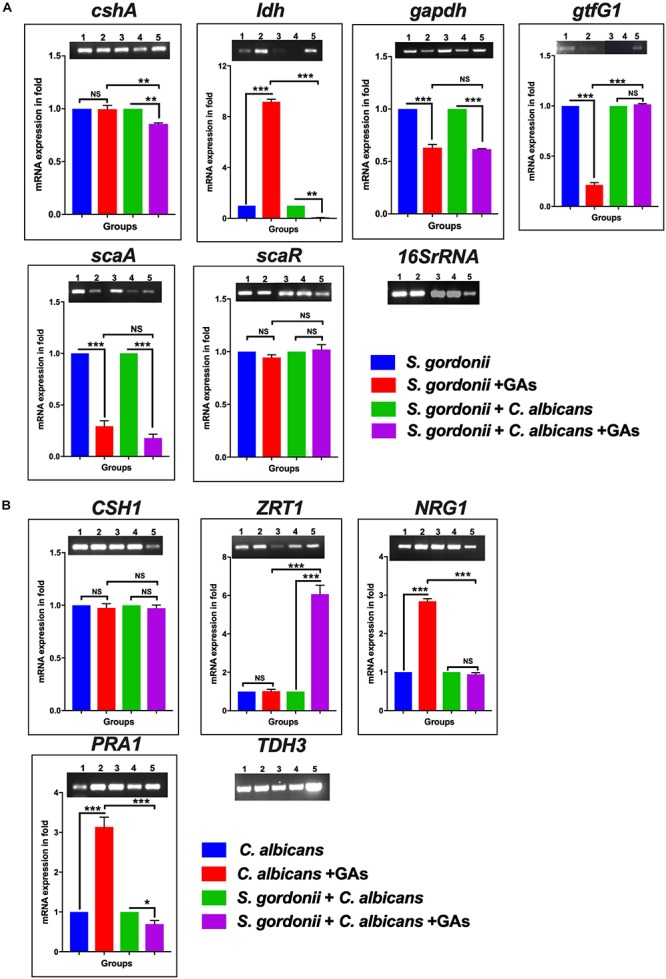
The mRNA expression level of biofilm genes as determined by RT-PCR. **(A)** Representative semi-quantitative mRNA expression profile for streptococcal primers showing the amplicons of mono-species and dual-species biofilms. (1) *S. gordonii*, (2) *S. gordonii* + GAs, (3) *S. gordonii* + *C. albicans*, (4) *S. gordonii* + (*C. albicans* + GAs, (5) Positive PCR control (gDNA used as template). Bar graphs represent the densitometry analysis of respective genes and a constant level of expression of 16S rRNA. **(B)** Representative semiquantitative mRNA expression profile for candida primers showing the amplicons of mono-species and dual-species biofilms. (1) *C. albicans*, (2) *C. albicans* + GAs, (3) *S. gordonii* + *C. albicans*, (4) *S. gordonii* + *C. albicans* + GAs, (5) Positive PCR control (gDNA as template). Bar graph represents the densitometry analysis of respective genes and a constant level of expression of *TDH3*. The results represent means ± standard deviations for three independent experiments. NS, not significant, ^∗^*p* < 0.05, ^∗∗^*p* < 0.01, ^∗∗∗^*p* < 0.001. *P* values were obtained by one-way ANOVA followed by Tukey’s multiple comparison test.)

Few studies have described differential expressions of genes during *S. gordonii* (Gilmore et al., 2003) or *Streptococci* + *C. albicans* dual-species biofilm growth (Dutton et al., 2016). To determine if some of these genes are affected by GAs treatment, a semi-quantitative RT-PCR analysis was used to examine variation in the expression of genes related to biofilm formation, i.e., *cshA, ldh, gapdh, gftG1, scaA*, and *scaR* for *S. gordonii* and *CSH1*, *ZRT1, NRG1*, and *PRA1*, for *C. albicans*. Treatment with GAs significantly reduced the expression of genes, including *scaA, gapdh*, and *gtfG1* in *S. gordonii* mono-species biofilms, whereas, in dual-species biofilms, *scaA*, *ldh*, and *cshA* were reduced in their expression when compared with their respective controls (Figure 5A). Interestingly, the expression of *ldh* was enhanced ninefold in GAs treated *S. gordonii* mono-species biofilms but not in dual-species biofilms (Figure 5A). In mono-species biofilms of *C. albicans*, the expression of *NRG1* was increased twofold in GAs treated samples compared to the untreated control (Figure 5B). No change was observed for *NRG1* and *CSH1* in GAs treated dual biofilms (Figure 5B and Supplementary Figure S3). The expression of *PRA1* was increased twofold in GAs exposed *C. abicans* mono-species biofilms, whereas, in dual-species biofilms, the expression of *PRA1* was decreased in GAs treated biofilms. In contrast, *ZRT1*, the regulator of *PRA1*, was overexpressed about fivefold in dual-species biofilms in the presence of GAs, but not in the GAs-exposed *C. albicans* mono-species biofilms.

### Inhibition of GAPDH Activity by GAs

**FIGURE 6:**
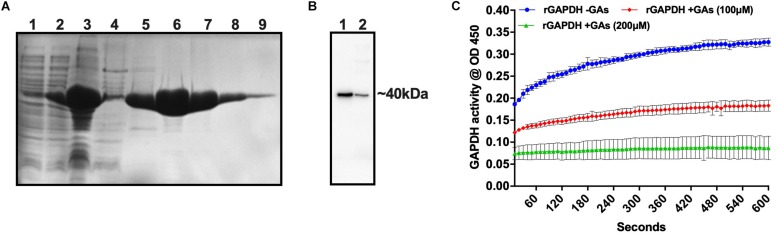
Purification of *S. gordonii* rGAPDH and determination of its enzyme activity. **(A)** SDS-PAGE gel showing purified fractions of rGAPDH protein from *E. coli* cell lysates. (1) Uninduced whole cell lysate, (2) Induced whole cell lysate, (3) French pressed cell lysate, (4) Unbound fraction, 5 to 9–100, 150, 200, 250, 300, and 350 mM imidazole eluted fractions, respectively. **(B)** Western blot of rGAPDH protein against anti-GAPDH antibody (anti rabbit *S. gordonii* GAPDH). Lanes, 1 and 2 – Purified rGAPDH protein at two different concentrations (3 and 1 μg). **(C)** Measurement of GAPDH activity in the presence and absence of GAs (treated, 200 μm equal to ∼160 μg/mL and 100 μm equal to ∼80 μg/mL).

Glyceraldehyde-3-phosphate dehydrogenase from streptococcal species is involved in pathogenesis and biofilm formation. Also, GAs treatment of *S. gordonii* mono-species or dual-species biofilms showed a reduction in its, gene expression. To assess the potential inhibitory activity of GAs against the GAPDH from *S. gordonii*, we cloned the gene, overexpressed and purified the rGAPDH protein using the *E. coli* expression system (Figure 6A). The purified rGAPDH migrated at an apparent molecular weight of ∼40 kDa and reacted to polyclonal anti-*Sg*GAPDH antibody (Figure 6B). We next tested the effect of GAs (100 and 200 μM) against the purified rGAPDH protein (0.1 μM). The assay depends on the conversion of glyceraldehyde-3-phosphate to 1,3-diphosphoglycerate by GAPDH enzyme in the presence of NAD. Interestingly, GAs appears to bind to GAPDH and block its enzyme activity in a dose-dependent manner. At 200 μM concentration, GAs block the activity of GAPDH completely when compared to the reaction without GAs where it shows strong enzyme activity (Figure 6C).

## Discussion

Microbial infection in the oral cavity of humans is biofilm-associated, where a significant proportion of infection was mixed biofilms. *S. gordonii*, an early colonizer of the oral cavity, forms an adhering biofilm on oral surfaces via cell surface adhesins (Bamford et al., 2009), leads to stable colonization in the oral cavity, and also attaches to *C. albicans* hyphae via protein-protein interactions (Holmes et al., 1996). In addition, *S. gordonii* colonization on the tooth surface allows other microbes to adhere and develop mixed biofilms such as dental caries, which is the most prevalent human oral disease, especially among the children. We have investigated the *S. gordonii* mono-species and *S. gordonii – C. albicans* dual-species biofilms and their inhibition by gymnemic acids (GAs) *in vitro.* GAs, a medicinal plant-derived small molecule, was shown to prevent *C. albicans* yeast-to-hypha transition and hyphal growth without affecting its viability or yeast growth rate (Vediyappan et al., 2013). However, GAs’ effect on bacterial and or bacterial-fungal mixed biofilms are unknown. GAs are a family of triterpenoid saponin compounds which are the major active principles of *G. sylvestre* plant leaves. The extract of this plant is widely used for its various medicinal properties, including lowering blood glucose activity in diabetic patients and reducing obesity (Porchezhian and Dobriyal, 2003; Leach, 2007; Zuniga et al., 2017).

Antibiofilm efficacy of GAs was investigated in terms of CV staining and eDNA reduction in the saliva-coated microtiter wells, where the results were found to be significant compared to untreated controls. This is the first report to provide evidence that the GAs shows antibiofilm efficacy against both mono-species and dual-species biofilms of *S. gordonii* and *C. albicans*. The microbial biofilms are protected by self-produced exopolysaccharides (EPS). EPS are generally made up of different types of polysaccharides, proteins, glycoproteins, glycolipids, and eDNA. The importance of eDNA release during early stages of biofilm is to preserve the structural firmness, enhancing the mixed biofilm and protection against antimicrobial agents (Mulcahy et al., 2008; Jack et al., 2015; Jung et al., 2017). Therefore, reduction of eDNA accumulation and other components could substantively diminish the development of biofilm formation. As such, we found that GAs was able to reduce a significant amount of eDNA in both mono-species and dual-species biofilms (Figures 1, 2).

It was reported earlier that *S. gordonii* cells form surface fibrils, which have multiple properties like cell surface hydrophobicity, co-aggregate with other oral bacteria, saliva-coated hydroxyapatite (sHA) and bind to host fibronectin (McNab et al., 1996; Back et al., 2017). These results emphasize that fibril-mediated attachment is the critical factor for the initial oral colonization for Streptococci. In the present study, we observed an extracellular nanofibrillar-mediated attachment of *S. gordonii* cells to sHA by SEM. Interestingly, these nanofibrils were not peritrichous as previously reported (McNab et al., 1999) and instead, the scattered fibrils were attached to neighboring streptococci cells, sHA substratum and to *C. albicans* hyphae (Figures 3, 4A–D) confirming its role in adherence. To our surprise, synthesis of these fibrils was abolished in the GAs treated *S. gordonii* biofilms. These fibrils could be related to EPS and we believe GAs might be affecting their synthesis and/or their incorporation into the biofilms. One of the unexpected findings of *S. gordonii–C. albicans* mixed biofilms was the formation of short fibrils from the *C. albicans* hyphae (Figures 4A,B). These fibrils show attachment to neighboring hypha and to the sHA substratum. This shows that there is an enhanced mutual synergism between these two microbes. However, in GAs treated mixed biofilms, these fibrils were absent (Figure 3L) and significant inhibition of biofilms was found. Djaczenko and Cassone (1972) have reported the presence of fimbriae in *C. albicans* yeast cells and known to contain mannosylated glycoprotein (Yu et al., 1994). We believe the fibrils that we observe in hyphae could be different from the fimbriae described above. For example, the fimbriae reported by Djaczenko and Cassone (1972) were found on the surface of “yeast cells” grown on agar plates for several days. These fimbriae are short and continuous throughout the cell surface of mother yeast cells but very little on the daughter cells.

In contrast, our results show the fibrils are discontinuous and found only from hyphae of *S. gordonii–C. albicans* co-cultured biofilms where they have close contacts with abiotic or biotic surfaces (Figure 4). These fibrils were not observed in biofilms grown in the presence of GAs, suggesting that GAs can prevent adhesive fibrils, in part, by inhibiting its synthesis and or hyphae associated adhesive proteins.

To understand the mechanisms of biofilm inhibition by GAs, we determined the expression of selected genes that have predicted roles in the growth of *S. gordonii* and *C. albicans* biofilms (Gilmore et al., 2003; Dutton et al., 2016). EPSs are the core parts for the assembly and maintenance of biofilm architectural integrity in the oral cavity. The oral streptococci produce glucosyltransferase enzymes, Gtfs, that split and use glucose from extracellular sucrose to synthesize glucans, which helps the streptococci adhere to the tooth surface and to the surfaces of other oral microbes. *S. gordonii*, the primary colonizer of the oral cavity, produces *gtfG1* (Vickerman et al., 1997). The RT-PCR analysis of *S. gordonii* biofilm cells shows basal level expression of *gtfG1*. However, GAs treatment reduced its expression, signifying the inhibitory potential of biofilm glucan by GAs (Figure 5A). This result agrees with SEM data where the *S. gordonii* biofilms treated with GAs show absence of adhesive fibrils when compared to the control biofilm where the fibrils can be seen between the biofilms cells and on the sHA (Figures 3D,J). The other roles of Gtfs include glycosylation of adhesive proteins such as GspB of *S. gordonii* and Fap1 of *Streptococcus parasanguinis* (Zhu et al., 2015). GAs are known to bind several proteins, including glucose transporter (Wang et al., 2014), taste receptors T1R2/T1R3 (Sanematsu et al., 2014), and Liver X-receptor (LXR) that regulates lipid metabolism in the liver (Renga et al., 2015). It has been reported that administration of GAs containing fraction, GS4, decreased the glycosylated hemoglobin (HbA1c) and glycosylated plasma protein in diabetic patients (Baskaran et al., 1990), and a similar mechanism may occur in microbial biofilms. Bacterial Gtfs play a critical role in enhancing the accumulation of *C. albicans* cells during mixed biofilm growths (Ellepola et al., 2017). GAs may affect the polysaccharide synthesis pathway in *S. gordonii* biofilms, through a reduced *gtfG1* expression and or its enzyme activity. Further, Gtfs use metal co-factor Mn^2+^ for enzyme catalytic activity (Zhu et al., 2015) and the downregulation of *scaA*, the gene that encodes Mn^2+^ binding lipoprotein, in GAs treated *S. gordonii* mono-species as well as dual-species biofilms (Figure 5) may also contribute to the reduction of adhesive fibrils/polysaccharides. For growth and survival in the human host, *S. gordonii* will have to acquire Mn^2+^ with the help of ScaA, a prominent surface antigen. It has been shown that inactivation of *scaA* gene resulted in both impaired growth of cells and >70% inhibition of Mn^2+^ uptake (Kolenbrander et al., 1998).

Oral bacteria, including *S. gordonii*, can sense the redox status of the biofilm niche and respond accordingly. Among the genes examined for differential expression in biofilms, we found lactate dehydrogenase (*ldh*) is one of the highly upregulated genes in GAs treated biofilms of *S. gordonii* (Figure 5). The ldh enzyme interconverts pyruvate into lactate and back, as it converts NADH to NAD and back. In GAs treated *S. gordonii*, ldh may be converting lactate into pyruvate as the *gapdh* mRNA is downregulated in GAs treated mono-species or mixed biofilms of *S. gordonii* but not in *C. albicans*. GAPDH uses NAD during glycolytic activity and the reduced amount of GAPDH may lead to the accumulation of NAD, which in turn activates the overexpression of *ldh* through a redox-sensing system (Bitoun and Wen, 2016). To determine if GAs has any effect on GAPDH enzyme activity, we cloned the *gapdh* gene from *S. gordonii*, overexpressed in *E. coli*, and tested the purified rGAPDH with or without GAs. We found the inhibition of rGAPDH enzyme activity in a dose-dependent manner (Figure 6). GA was shown to inhibit rabbit GAPDH enzyme activity (Izutani et al., 2005). Maeda et al. (2004a, b) have showed that oral streptococcal (e.g., *S. oralis, S. gordonii*) cell surface-associated GAPDH binds to the long fimbriae (FimA) of *P. gingivalis* and play a role in the development of oral polymicrobial biofilms (Kuboniwa et al., 2017). In addition to glycolytic function, GAPDH is also a moonlighting protein and known to carry out multiple functions (Sirover, 2017). It is worth mentioning that natural products (anacardic acid and curcumin) have been shown to bind and inhibit *Streptococcus pyogenes* GAPDH activity. GAPDH is a major virulence factor (Gomez et al., 2019), and the GAPDH serves as a drug target for other pathogens (Freitas et al., 2009) as well. GAs appear to impact *S. gordonii* GAPDH both at the transcriptional and translational level and could account, at least partially, for the observed inhibition of *S. gordonii* growth and or biofilm. Comparison of amino acid sequences of both *S. gordonii* and *C. albicans* GAPDH revealed about 50% similarity, leaving open the possibility that GAs impact on them could be different. In fact, the expression of GAPDH gene in *C. albicans* (*TDH3*) biofilms grown in the presence or absence of GAs is not affected (Figure 5B, *TDH3* RT-PCR bands). However, GAs impact on *C. albicans* GAPDH (Tdh3) enzyme activity and its role in biofilms can’t be ruled out and remains to be determined. Global gene expression and biochemical analyzes are necessary steps to reveal the mechanism(s) of GAs-mediated inhibition of *S. gordonii* mono-species and dual-species biofilms.

Among the genes examined in *C. albicans* mono-species or dual-species biofilms, *NRG1, PRA1*, and *ZRT1* are the most differentially expressed. It is well known from the literature that Nrg1 of *C. albicans* is a DNA binding protein that represses its filamentous growth (Braun et al., 2001). GAs treatment shows a significant increase of *NRG1* mRNA expression in *C. albicans* biofilms compared to control biofilms (Figure 5B), which may correspond to the observed yeast or pseudohyphal growth forms of *C. albicans* mono-species biofilm (Figure 3). However, no change of *NRG1* expression level was observed in dual-species biofilms, yet their biofilm growth was inhibited, underscoring the unknown regulatory mechanism in the GAs treated dual-species biofilms. *C. albicans* sequesters environmental zinc through a secreted protein, the pH-regulated antigen 1 (Pra1) and transports it through the membrane transporter (Zrt1) for its invasive growth in the host (Citiulo et al., 2012). *C. albicans* has biphasic mechanisms for its environmental and cellular zinc homeostasis and Pra1 expresses when cells are at pH 7 and above or at zinc limitation (Crawford et al., 2018; Wilson, 2019). Kurakado et al. (2018) have also reported that the hypha-related Pra1 and Zrt1 play a major regulatory role *C. albicans* biofilm formation through zinc homeostasis. GAs treatment to *C. albicans* mono-species biofilm appears to cause zinc limitation and or change in cellular pH, which could be altered when grown with *S. gordonii* as mixed-species biofilms (Figure 5B).

Our understanding about mixed species biofilms in caries pathogenesis is still in its infancy (Metwalli et al., 2013). It was well known that from various host defense factors, microbes in mixed biofilms act synergistically for their survival (Morales and Hogan, 2010; Xu et al., 2014a). Great attention is needed on mitis group streptococci (*S. gordonii*, *S. oralis*, *Streptococcus mitis*, *S. parasanguinis*, and *Streptococcus sanguinis*), which form multispecies biofilms when aggregating with other bacterial and fungal species (Xu et al., 2014b). These oral microbial infections pose a significant threat to public health, as many pathogenic bacteria readily develop resistance to multiple antibiotics and form biofilms with additional protection from antibiotic treatment (Lebeaux et al., 2014). Currently available antimicrobial agents were most effective at drastically reducing the cell viability, rather than reducing the virulence via inhibiting the biofilm growth. For instance, fluoride is a proven agent for caries prophylaxis; however, excess use of fluoride causes fluorosis and hardening of cartilage. Also, these synthetic antimicrobial agents lead to negative effects in the gastrointestinal system and several other side effects. We are in need of efficient antimicrobial agent which inhibit biofilm formation, while at the same time the agent should not exert selective pressure over oral microbiome.

Recently, many studies have targeted medicinal plants in finding effective anticaries agents (Islam et al., 2008; Yang et al., 2017; Gartika et al., 2018; Henley-Smith et al., 2018). Medicinal plants have been used to prevent and treat microbial diseases since ancient times, which can target several antigens or pathways of the pathogens for inhibition without adverse effects. Earlier studies on medicinal plant extracts described biofilm inhibition by hindering hydrophobic properties of *S. mutans* (Nostro et al., 2004; Khan et al., 2012). Any antimicrobial agent that reduces or hinders interactions/attachment represents a novel strategy to overcome oral infection. Interestingly, our GAs treatment shows a significant reduction in both mono-species and dual-species biofilms and appear to act via more than one mechanism. GAs affect the transcription of *S. gordonii gapdh* and its enzyme activity in addition to *gtfG1*, which is involved in glucan polysaccharide synthesis. Further, GAs are able to curtail the development of nanofibrils that mediate cell-cell and substrate adhesion both in *S. gordonii* and *C. albicans*. In summary, our findings offer an anti-virulence approach for preventing mixed oral biofilms and by further optimization, and natural products have high potential as a useful source for developing mixed biofilm inhibitors.

## Data Availability Statement

All datasets generated for this study are included in the manuscript/Supplementary Files.

## Ethics Statement

The studies involving human participants were reviewed and approved by Institutional Review Board, Kansas State University. The patients/participants provided their written informed consent to participate in this study.

## Author Contributions

GV designed the study. RV and GV conducted the experiments, analyzed the data, and wrote the manuscript.

## Conflict of Interest

The authors declare that the research was conducted in the absence of any commercial or financial relationships that could be construed as a potential conflict of interest.

## References

[B1] Back C. R. Sztukowska M. N. Till M. Lamont R. J. Jenkinson H. F. Nobbs A. H. (2017). The *Streptococcus gordonii* adhesin CshA protein binds host fibronectin via a catch-clamp mechanism. *J. Biol. Chem.* 292 1538–1549. 10.1074/jbc.M116.760975 27920201 PMC5290933

[B2] Bamford C. V. D’mello A. Nobbs A. H. Dutton L. C. Vickerman M. M. Jenkinson H. F. (2009). *Streptococcus gordonii* modulates *Candida albicans* biofilm formation through intergeneric communication. *Infect. Immun.* 77 3696–3704. 10.1128/IAI.00438-09 19528215 PMC2737996

[B3] Baskaran K. Kizar Ahamath B. Radha Shanmugasundaram K. Shanmugasundaram E. R. (1990). Antidiabetic effect of a leaf extract from *Gymnema sylvestre* in non-insulin-dependent diabetes mellitus patients. *J. Ethnopharmacol.* 30 295–300. 2259217 10.1016/0378-8741(90)90108-6

[B4] Bitoun J. P. Wen Z. T. (2016). Transcription factor Rex in regulation of pathophysiology in oral pathogens. *Mol. Oral Microbiol.* 31 115–124. 10.1111/omi.12114 26172563 PMC4713358

[B5] Brassard J. Gottschalk M. Quessy S. (2004). Cloning and purification of the *Streptococcus suis* serotype 2 glyceraldehyde-3-phosphate dehydrogenase and its involvement as an adhesin. *Vet. Microbiol.* 102 87–94. 10.1016/j.vetmic.2004.05.008 15288930

[B6] Braun B. R. Kadosh D. Johnson A. D. (2001). NRG1, a repressor of filamentous growth in *Candida albicans*, is down-regulated during filament induction. *EMBO J.* 20 4753–4761. 10.1093/emboj/20.17.4753 11532939 PMC125265

[B7] Citiulo F. Jacobsen I. D. Miramon P. Schild L. Brunke S. Zipfel P. (2012). *Candida albicans* scavenges host zinc via Pra1 during endothelial invasion. *PLoS Pathog.* 8:e1002777. 10.1371/journal.ppat.1002777 22761575 PMC3386192

[B8] Crawford A. C. Lehtovirta-Morley L. E. Alamir O. Niemiec M. J. Alawfi B. Alsarraf M. (2018). Biphasic zinc compartmentalisation in a human fungal pathogen. *PLoS Pathog.* 14:e1007013. 10.1371/journal.ppat.1007013 29727465 PMC5955600

[B9] Di Fabio G. Romanucci V. Zarrelli M. Giordano M. Zarrelli A. (2013). C-4 gem-dimethylated oleanes of *Gymnema sylvestre* and their pharmacological activities. *Molecules* 18 14892–14919. 10.3390/molecules181214892 24304585 PMC6269971

[B10] Diaz P. I. Xie Z. Sobue T. Thompson A. Biyikoglu B. Ricker A. (2012). Synergistic interaction between *Candida albicans* and commensal oral streptococci in a novel in vitro mucosal model. *Infect. Immun.* 80 620–632. 10.1128/IAI.05896-11 22104105 PMC3264323

[B11] Djaczenko W. Cassone A. (1972). Visulization of new ultrastructural components in the cell wall of *Candida albicans* with fixatives containing TAPO. *J. Cell Biol.* 52 186–190. 10.1083/jcb.52.1.186 4550098 PMC2108676

[B12] Dongari-Bagtzoglou A. Kashleva H. Dwivedi P. Diaz P. Vasilakos J. (2009). Characterization of mucosal *Candida albicans* biofilms. *PLoS One* 4:e7967. 10.1371/journal.pone.0007967 19956771 PMC2776351

[B13] Dutton L. C. Nobbs A. H. Jepson K. Jepson M. A. Vickerman M. M. Aqeel Alawfi S. (2014). O-mannosylation in *Candida albicans* enables development of interkingdom biofilm communities. *mBio* 5:e911-14. 10.1128/mBio.00911-14 24736223 PMC3993854

[B14] Dutton L. C. Paszkiewicz K. H. Silverman R. J. Splatt P. R. Shaw S. Nobbs A. H. (2016). Transcriptional landscape of trans-kingdom communication between *Candida albicans* and *Streptococcus gordonii*. *Mol. Oral Microbiol.* 31 136–161. 10.1111/omi.12111 26042999 PMC4670286

[B15] Ellepola K. Liu Y. Cao T. Koo H. Seneviratne C. J. (2017). Bacterial GtfB augments *Candida albicans* accumulation in cross-kingdom biofilms. *J. Dent. Res.* 96 1129–1135. 10.1177/0022034517714414 28605597 PMC5582686

[B16] Erlandsen S. L. Kristich C. J. Dunny G. M. Wells C. L. (2004). High-resolution visualization of the microbial glycocalyx with low-voltage scanning electron microscopy: dependence on cationic dyes. *J. Histochem. Cytochem.* 52 1427–1435. 10.1369/jhc.4a6428.2004 15505337 PMC3957825

[B17] Freitas R. F. Prokopczyk I. M. Zottis A. Oliva G. Andricopulo A. D. Trevisan M. T. (2009). Discovery of novel *Trypanosoma cruzi* glyceraldehyde-3-phosphate dehydrogenase inhibitors. *Bioorg. Med. Chem.* 17 2476–2482. 10.1016/j.bmc.2009.01.079 19254846

[B18] Gartika M. Pramesti H. T. Kurnia D. Satari M. H. (2018). A terpenoid isolated from *Sarang semut* (Myrmecodia pendans) bulb and its potential for the inhibition and eradication of *Streptococcus mutans* biofilm. *BMC Complement. Altern. Med.* 18:151. 10.1186/s12906-018-2213-x 29739390 PMC5941495

[B19] Gilmore K. S. Srinivas P. Akins D. R. Hatter K. L. Gilmore M. S. (2003). Growth, development, and gene expression in a persistent *Streptococcus gordonii* biofilm. *Infect. Immun.* 71 4759–4766. 10.1128/iai.71.8.4759-4766.2003 12874358 PMC166047

[B20] Gomez S. Querol-Garcia J. Sanchez-Barron G. Subias M. Gonzalez-Alsina A. Franco-Hidalgo V. (2019). The antimicrobials anacardic acid and curcumin are not-competitive inhibitors of gram-positive bacterial pathogenic Glyceraldehyde-3-phosphate dehydrogenase by a mechanism unrelated to human C5a anaphylatoxin binding. *Front. Microbiol.* 10:326. 10.3389/fmicb.2019.00326 30863383 PMC6400076

[B21] Harriott M. M. Noverr M. C. (2009). *Candida albicans* and *Staphylococcus aureus* form polymicrobial biofilms: effects on antimicrobial resistance. *Antimicrob. Agents Chemother.* 53 3914–3922. 10.1128/AAC.00657-09 19564370 PMC2737866

[B22] Harriott M. M. Noverr M. C. (2011). Importance of *Candida*-bacterial polymicrobial biofilms in disease. *Trends Microbiol.* 19 557–563. 10.1016/j.tim.2011.07.004 21855346 PMC3205277

[B23] Henley-Smith C. J. Botha F. S. Hussein A. A. Nkomo M. Meyer D. Lall N. (2018). Biological activities of heteropyxis natalensis against micro-organisms involved in oral infections. *Front. Pharmacol.* 9:291. 10.3389/fphar.2018.00291 29692723 PMC5903190

[B24] Holmes A. R. Mcnab R. Jenkinson H. F. (1996). *Candida albicans* binding to the oral bacterium *Streptococcus gordonii* involves multiple adhesin-receptor interactions. *Infect. Immun.* 64 4680–4685. 8890225 10.1128/iai.64.11.4680-4685.1996 PMC174431

[B25] Hwang G. Liu Y. Kim D. Li Y. Krysan D. J. Koo H. (2017). *Candida albicans* mannans mediate *Streptococcus mutans* exoenzyme GtfB binding to modulate cross-kingdom biofilm development in vivo. *PLoS Pathog.* 13:e1006407. 10.1371/journal.ppat.1006407 28617874 PMC5472321

[B26] Islam B. Khan S. N. Haque I. Alam M. Mushfiq M. Khan A. U. (2008). Novel anti-adherence activity of mulberry leaves: inhibition of *Streptococcus mutans* biofilm by 1-deoxynojirimycin isolated from *Morus alba*. *J. Antimicrob. Chemother.* 62 751–757. 10.1093/jac/dkn253 18565974

[B27] Izutani Y. Murai T. Imoto T. Ohnishi M. Oda M. Ishijima S. (2005). Gymnemic acids inhibit rabbit glyceraldehyde-3-phosphate dehydrogenase and induce a smearing of its electrophoretic band and dephosphorylation. *FEBS Lett.* 579 4333–4336. 10.1016/j.febslet.2005.06.070 16054141

[B28] Jack A. A. Daniels D. E. Jepson M. A. Vickerman M. M. Lamont R. J. Jenkinson H. F. (2015). *Streptococcus gordonii* comCDE (competence) operon modulates biofilm formation with *Candida albicans*. *Microbiology* 161 411–421. 10.1099/mic.0.000010 25505189 PMC4314648

[B29] Jin H. Agarwal S. Agarwal S. Pancholi V. (2011). Surface export of GAPDH/SDH, a glycolytic enzyme, is essential for *Streptococcus pyogenes* virulence. *mBio* 2:e68-11. 10.1128/mBio.00068-11 21628503 PMC3104492

[B30] Jung C. J. Hsu R. B. Shun C. T. Hsu C. C. Chia J. S. (2017). AtlA mediates extracellular DNA release, which contributes to *Streptococcus mutans* biofilm formation in an experimental rat model of infective Endocarditis. *Infect. Immun.* 85:e252-17. 10.1128/IAI.00252-17 28674029 PMC5563578

[B31] Kassebaum N. J. Smith A. G. C. Bernabe E. Fleming T. D. Reynolds A. E. Vos T. (2017). Global, regional, and national prevalence, incidence, and disability-adjusted life years for oral conditions for 195 countries, 1990-2015: a systematic analysis for the global burden of diseases, injuries, and risk factors. *J. Dent. Res.* 96 380–387. 10.1177/0022034517693566 28792274 PMC5912207

[B32] Khan R. Adil M. Danishuddin M. Verma P. K. Khan A. U. (2012). *In vitro* and *in vivo* inhibition of *Streptococcus mutans* biofilm by *Trachyspermum ammi* seeds: an approach of alternative medicine. *Phytomedicine* 19 747–755. 10.1016/j.phymed.2012.04.004 22633847

[B33] Kim D. Sengupta A. Niepa T. H. Lee B. H. Weljie A. Freitas-Blanco V. S. (2017). *Candida albicans* stimulates *Streptococcus mutans* microcolony development via cross-kingdom biofilm-derived metabolites. *Sci. Rep.* 7:41332. 10.1038/srep41332 28134351 PMC5278416

[B34] Kolenbrander P. E. Andersen R. N. Baker R. A. Jenkinson H. F. (1998). The adhesion-associated sca operon in *Streptococcus gordonii* encodes an inducible high-affinity ABC transporter for Mn2+ uptake. *J. Bacteriol.* 180 290–295. 9440518 10.1128/jb.180.2.290-295.1998 PMC106884

[B35] Kuboniwa M. Houser J. R. Hendrickson E. L. Wang Q. Alghamdi S. A. Sakanaka A. (2017). Metabolic crosstalk regulates *Porphyromonas gingivalis* colonization and virulence during oral polymicrobial infection. *Nat. Microbiol.* 2 1493–1499. 10.1038/s41564-017-0021-6 28924191 PMC5678995

[B36] Kurakado S. Arai R. Sugita T. (2018). Association of the hypha-related protein Pra1 and zinc transporter Zrt1 with biofilm formation by the pathogenic yeast *Candida albicans*. *Microbiol. Immunol.* 62 405–410. 10.1111/1348-0421.12596 29704397

[B37] Leach M. J. (2007). *Gymnema sylvestre* for diabetes mellitus: a systematic review. *J. Altern. Complement. Med.* 13 977–983. 10.1089/acm.2006.6387 18047444

[B38] Lebeaux D. Ghigo J. M. Beloin C. (2014). Biofilm-related infections: bridging the gap between clinical management and fundamental aspects of recalcitrance toward antibiotics. *Microbiol. Mol. Biol. Rev.* 78 510–543. 10.1128/MMBR.00013-14 25184564 PMC4187679

[B39] Liu H. M. Kiuchi F. Tsuda Y. (1992). Isolation and structure elucidation of gymnemic acids, antisweet principles of *Gymnema sylvestre*. *Chem. Pharm. Bull.* 40 1366–1375. 10.1248/cpb.40.1366 1327559

[B40] Lo H. J. Kohler J. R. Didomenico B. Loebenberg D. Cacciapuoti A. Fink G. R. (1997). Nonfilamentous *Candida albicans* mutants are avirulent. *Cell* 90 939–949. 10.1016/s0092-8674(00)80358-x 9298905

[B41] Maeda K. Nagata H. Nonaka A. Kataoka K. Tanaka M. Shizukuishi S. (2004a). Oral streptococcal glyceraldehyde-3-phosphate dehydrogenase mediates interaction with *Porphyromonas gingivalis* fimbriae. *Microbes Infect.* 6 1163–1170. 10.1016/j.micinf.2004.06.005 15488735

[B42] Maeda K. Nagata H. Yamamoto Y. Tanaka M. Tanaka J. Minamino N. (2004b). Glyceraldehyde-3-phosphate dehydrogenase of *Streptococcus oralis* functions as a coadhesin for *Porphyromonas gingivalis* major fimbriae. *Infect. Immun.* 72 1341–1348. 10.1128/iai.72.3.1341-1348.2004 14977937 PMC355992

[B43] McNab R. Forbes H. Handley P. S. Loach D. M. Tannock G. W. Jenkinson H. F. (1999). Cell wall-anchored CshA polypeptide (259 kilodaltons) in *Streptococcus gordonii* forms surface fibrils that confer hydrophobic and adhesive properties. *J. Bacteriol.* 181 3087–3095. 10322009 10.1128/jb.181.10.3087-3095.1999 PMC93763

[B44] McNab R. Holmes A. R. Clarke J. M. Tannock G. W. Jenkinson H. F. (1996). Cell surface polypeptide CshA mediates binding of *Streptococcus gordonii* to other oral bacteria and to immobilized fibronectin. *Infect. Immun.* 64 4204–4210. 8926089 10.1128/iai.64.10.4204-4210.1996 PMC174357

[B45] Merritt J. H. Kadouri D. E. O’toole G. A. (2005). Growing and analyzing static biofilms. *Curr. Protoc. Microbiol.* 0 1:Unit–1B.1. 10.1002/9780471729259.mc01b01s00 18770545 PMC4568995

[B46] Metwalli K. H. Khan S. A. Krom B. P. Jabra-Rizk M. A. (2013). *Streptococcus mutans*, *Candida albicans*, and the human mouth: a sticky situation. *PLoS Pathog.* 9:e1003616. 10.1371/journal.ppat.1003616 24146611 PMC3798555

[B47] Morales D. K. Hogan D. A. (2010). *Candida albicans* interactions with bacteria in the context of human health and disease. *PLoS Pathog.* 6:e1000886. 10.1371/journal.ppat.1000886 20442787 PMC2861711

[B48] Mulcahy H. Charron-Mazenod L. Lewenza S. (2008). Extracellular DNA chelates cations and induces antibiotic resistance in *Pseudomonas aeruginosa* biofilms. *PLoS Pathog.* 4:e1000213. 10.1371/journal.ppat.1000213 19023416 PMC2581603

[B49] Nett J. E. Sanchez H. Cain M. T. Andes D. R. (2010). Genetic basis of *Candida* biofilm resistance due to drug-sequestering matrix glucan. *J. Infect. Dis.* 202 171–175. 10.1086/651200 20497051 PMC2880631

[B50] Nobile C. J. Mitchell A. P. (2006). Genetics and genomics of *Candida albicans* biofilm formation. *Cell Microbiol.* 8 1382–1391. 10.1111/j.1462-5822.2006.00761.x 16848788

[B51] Nostro A. Cannatelli M. A. Crisafi G. Musolino A. D. Procopio F. Alonzo V. (2004). Modifications of hydrophobicity, *in vitro* adherence and cellular aggregation of *Streptococcus* mutans by Helichrysum italicum extract. *Lett. Appl. Microbiol.* 38 423–427. 10.1111/j.1472-765x.2004.01509.x 15059215

[B52] Odds F. C. (1987). *Candida* infections: an overview. *Crit. Rev. Microbiol* 15 1–5. 3319417 10.3109/10408418709104444

[B53] O’Donnell L. E. Millhouse E. Sherry L. Kean R. Malcolm J. Nile C. J. (2015). Polymicrobial *Candida* biofilms: friends and foe in the oral cavity. *FEMS Yeast Res.* 15:fov077. 10.1093/femsyr/fov077 26298018

[B54] Porchezhian E. Dobriyal R. M. (2003). An overview on the advances of *Gymnema sylvestre*: chemistry, pharmacology and patents. *Pharmazie* 58 5–12. 12622244

[B55] Renga B. Festa C. De Marino S. Di Micco S. D’auria M. V. Bifulco G. (2015). Molecular decodification of gymnemic acids from *Gymnema sylvestre*. Discovery of a new class of liver X receptor antagonists. *Steroids* 96 121–131. 10.1016/j.steroids.2015.01.024 25668616

[B56] Ricker A. Vickerman M. Dongari-Bagtzoglou A. (2014). *Streptococcus gordonii* glucosyltransferase promotes biofilm interactions with *Candida albicans*. *J. Oral. Microbiol.* 6. 10.3402/jom.v6.23419 24490004 PMC3907680

[B57] Righolt A. J. Jevdjevic M. Marcenes W. Listl S. (2018). Global-, Regional-, and Country-level economic impacts of dental diseases in 2015. *J. Dent. Res.* 97 501–507. 10.1177/0022034517750572 29342371

[B58] Sanematsu K. Kusakabe Y. Shigemura N. Hirokawa T. Nakamura S. Imoto T. (2014). Molecular mechanisms for sweet-suppressing effect of gymnemic acids. *J. Biol. Chem.* 289 25711–25720. 10.1074/jbc.M114.560409 25056955 PMC4162174

[B59] Saputo S. Faustoferri R. C. Quivey R. G. Jr. (2018). A drug repositioning approach reveals that *Streptococcus mutans* is susceptible to a diverse range of established antimicrobials and nonantibiotics. *Antimicrob. Agents Chemother.* 62:e1674-17. 10.1128/AAC.01674-17 29061736 PMC5740335

[B60] Silverman R. J. Nobbs A. H. Vickerman M. M. Barbour M. E. Jenkinson H. F. (2010). Interaction of *Candida albicans* cell wall Als3 protein with *Streptococcus gordonii* SspB adhesin promotes development of mixed-species communities. *Infect. Immun.* 78 4644–4652. 10.1128/IAI.00685-10 20805332 PMC2976310

[B61] Sirover M. A. (2017). *Glyceraldehyde-3-Phosphate Dehydrogenase (GAPDH). The Quintessential Moonlighting Protein in Normal Cell Function and in Human Disease.* Cambridge, MA: Academic Press, 324.

[B62] Sivaprakasam C. Vijayakumar R. Arul M. Nachiappan V. (2016). Alteration of mitochondrial phospholipid due to the PLA2 activation in rat brains under cadmium toxicity. *Toxicol. Res.* 5 1680–1687. 10.1039/c6tx00201c 30090467 PMC6062122

[B63] Tati S. Davidow P. Mccall A. Hwang-Wong E. Rojas I. G. Cormack B. (2016). *Candida glabrata* binding to *Candida albicans* hyphae enables its development in oropharyngeal candidiasis. *PLoS Pathog.* 12:e1005522. 10.1371/journal.ppat.1005522 27029023 PMC4814137

[B64] Uppuluri P. Lin L. Alqarihi A. Luo G. Youssef E. G. Alkhazraji S. (2018). The Hyr1 protein from the fungus *Candida albicans* is a cross kingdom immunotherapeutic target for Acinetobacter bacterial infection. *PLoS Pathog.* 14:e1007056. 10.1371/journal.ppat.1007056 29746596 PMC5963808

[B65] Vediyappan G. Dumontet V. Pelissier F. D’enfert C. (2013). Gymnemic acids inhibit hyphal growth and virulence in *Candida albicans*. *PLoS One* 8:e74189. 10.1371/journal.pone.0074189 24040201 PMC3770570

[B66] Vediyappan G. Rossignol T. D’enfert C. (2010). Interaction of *Candida albicans* biofilms with antifungals: transcriptional response and binding of antifungals to beta-glucans. *Antimicrob. Agents Chemother.* 54 2096–2111. 10.1128/AAC.01638-09 20194705 PMC2863626

[B67] Vickerman M. M. Sulavik M. C. Nowak J. D. Gardner N. M. Jones G. W. Clewell D. B. (1997). Nucleotide sequence analysis of the *Streptococcus gordonii* glucosyltransferase gene, gtfG. *DNA Seq.* 7 83–95. 10.3109/10425179709020155 9063645

[B68] Wang Y. Dawid C. Kottra G. Daniel H. Hofmann T. (2014). Gymnemic acids inhibit sodium-dependent glucose transporter 1. *J. Agric. Food Chem.* 62 5925–5931. 10.1021/jf501766u 24856809

[B69] Wang Y. Yi L. Wu Z. Shao J. Liu G. Fan H. (2012). Comparative proteomic analysis of *Streptococcus suis* biofilms and planktonic cells that identified biofilm infection-related immunogenic proteins. *PLoS One* 7:e33371. 10.1371/journal.pone.0033371 22514606 PMC3326019

[B70] Wilson D. (2019). *Candida albicans*. *Trends Microbiol.* 27 188–189. 10.1016/j.tim.2018.10.010 30551845

[B71] Xu H. Jenkinson H. F. Dongari-Bagtzoglou A. (2014a). Innocent until proven guilty: mechanisms and roles of *Streptococcus*-*Candida* interactions in oral health and disease. *Mol. Oral Microbiol.* 29 99–116. 10.1111/omi.12049 24877244 PMC4238848

[B72] Xu H. Sobue T. Thompson A. Xie Z. Poon K. Ricker A. (2014b). Streptococcal co-infection augments *Candida* pathogenicity by amplifying the mucosal inflammatory response. *Cell Microbiol.* 16 214–231. 10.1111/cmi.12216 24079976 PMC3956708

[B73] Xu Y. Kreth J. (2013). Role of LytF and AtlS in eDNA release by *Streptococcus gordonii*. *PLoS One* 8:e62339. 10.1371/journal.pone.0062339 23638042 PMC3634736

[B74] Yang H. Li K. Yan H. Liu S. Wang Y. Huang C. (2017). High-performance therapeutic quercetin-doped adhesive for adhesive-dentin interfaces. *Sci. Rep.* 7:8189. 10.1038/s41598-017-08633-3 28811592 PMC5558009

[B75] Yu L. Lee K. K. Ens K. Doig P. C. Carpenter M. R. Staddon W. (1994). Partial characterization of a *Candida albicans* fimbrial adhesin. *Infect. Immun.* 62 2834–2842. 8005673 10.1128/iai.62.7.2834-2842.1994 PMC302889

[B76] Zhu F. Zhang H. Wu H. (2015). Glycosyltransferase-mediated sweet modification in oral *Streptococci*. *J. Dent. Res.* 94 659–665. 10.1177/0022034515574865 25755271 PMC4502785

[B77] Zuniga L. Y. Gonzalez-Ortiz M. Martinez-Abundis E. (2017). Effect of *Gymnema sylvestre* administration on metabolic syndrome, insulin sensitivity, and insulin secretion. *J. Med. Food* 20 750–754. 10.1089/jmf.2017.0001 28459647

