# Supplementary material for: Gymnemic Acids Inhibit Adhesive Nanofibrillar Mediated Streptococcus gordonii–Candida albicans Mono-Species and Dual-Species Biofilms
Source: Front Microbiol. 2019 Oct 11;10:2328. doi: 10.3389/fmicb.2019.02328 (PMC6797559; doi:10.3389/fmicb.2019.02328)
Supplement: Supplementary file 1 [file Data_Sheet_1.pdf]

Supplementary information

Gymnemic Acids Inhibit Adhesive Nanofibrillar Mediated *Streptococcus gordonii-Candida albicans* Mono-species and Dual-Species Biofilms

Raja Veerapandian and Govindsamy Vediyappan*

*Corresponding author

Figure S1. Restriction endonuclease analysis of GAPDH constructs cloned in pET28b vector

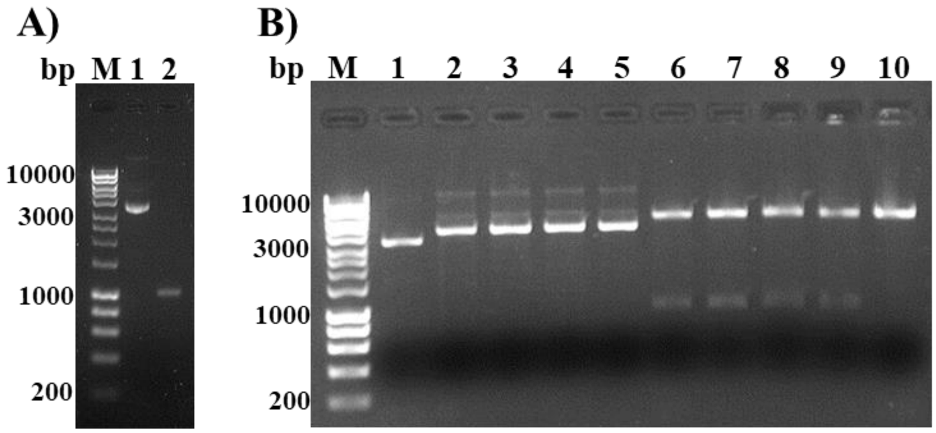

Legends: A) Agarose gel showing pET28b vector and PCR amplified GAPDH gene, M- 1Kb marker, 1- pET28b vector undigested, 2- PCR purified *S. gordonii* GAPDH gene (1008bp).
B) Restriction endonuclease analysis (REA) of GAPDH constructs in pET28b vector, M - 1Kb Marker (Gene Ruler, 1 kb, NEB), 1- pET28b vector undigested, 2 to 5 – Undigested pET28b GAPDH constructs (4 colonies), 6 to 9 – Double digested pET28b GAPDH constructs, 10 – Double digested pET28b vector (5368bp). Plasmid DNA #2 was used for DNA sequencing and GAPDH overexpression in *E. coli*.

Figure S2. Sequence analysis of pET28b-GAPDH construct (#2)

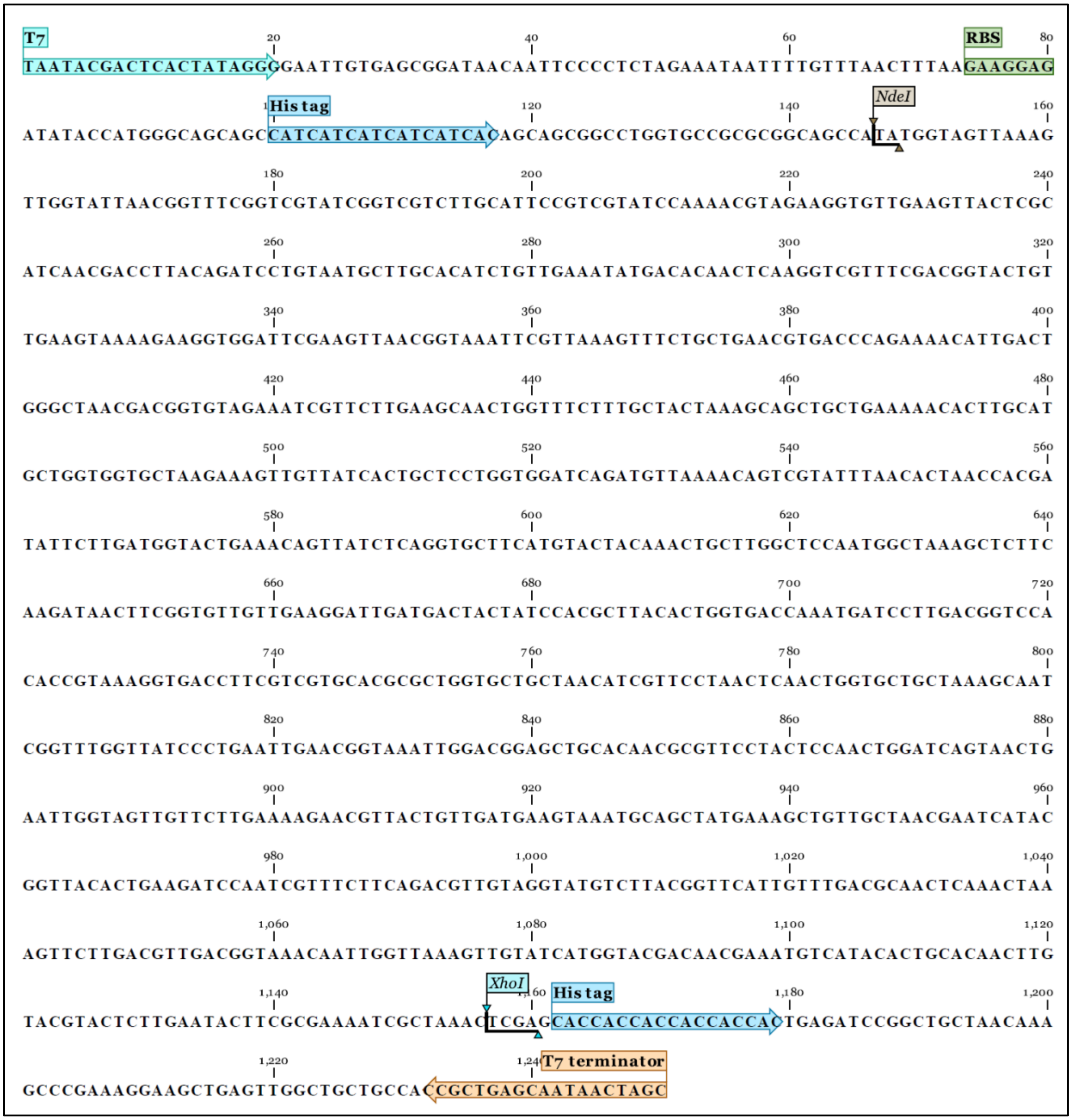

Legends: The pET28b-GAPDH construct (#2) was sequenced in both forward and reverse directions by sanger sequencing. The resulting sequence was assembled using CAP3 Sequence Assembly Program (http://doua.prabi.fr/software/cap3) and the vector sequences were identified by VecScreen (https://www.ncbi.nlm.nih.gov/tools/vecscreen/). Sequence graphics were done using CLC genomics workbench 12.

Figure S3. The mRNA expression level of *MNT1* and *MNT2* by RT-PCR

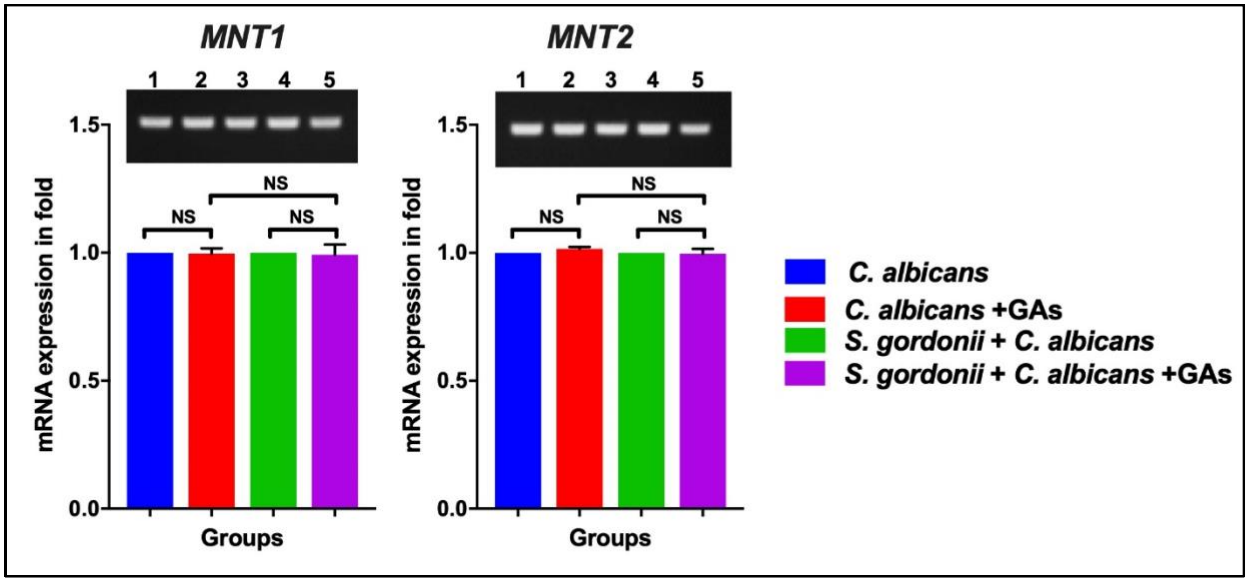

Legends: Representative semiquantitative mRNA expression profile for MNT1 and MNT2 amplicons of mono-species and dual-species biofilms. 1. *C. albicans*, 2. *C. albicans* + GAs, 3. *S. gordonii* + *C. albicans*, 4. *S. gordonii* + *C. albicans* + GAs, 5. Positive PCR control (gDNA as template). Bar graph represents the densitometry analysis of respective genes. The results represent means ± standard deviations for three independent experiments. NS-not significant.

Semi-quantitative RT-PCR Primers used

Gene name	Description	Direction	Sequence (5'-3')	Product Size (bp)
MNT1	Mannosyl transferase 1	Forward	CTGGTGAAGGTGGTAGTGATG	296
		Reverse	CCATGGAGGATATGACCAATGT	
MNT2	Mannosyl transferase 2	Forward	GCAGTTATCTGGCTGATCCTAAT	264
		Reverse	CTTGTTTCTCTTGTTGCTGTGG	
